# Incorporating natural language processing-derived features from pathology reports into machine learning models to predict differentiated thyroid cancer recurrence

**DOI:** 10.3389/fendo.2026.1939077

**Published:** 2026-10-21

**Authors:** Denise T. Lee, Steven Yen, Grace Deng, Yehia Elkersh, Andrew M. Thomas, Usha Govindarajulu, Michael L. Marin, Girish N. Nadkarni, David S. Matteson

**Affiliations:** 1 Department of Surgery, Icahn School of Medicine at Mount Sinai, New York, NY, United States; 2 Windreich Department of Artificial Intelligence and Human Health, Icahn School of Medicine at Mount Sinai, New York, NY, United States; 3 Department of Statistics and Data Science, Cornell University, Ithaca, NY, United States; 4 Department of Statistics and Actuarial Science, University of Iowa, Iowa City, IA, United States; 5 Center for Biostatistics, Department of Population Health Science and Policy, Icahn School of Medicine at Mount Sinai, New York, NY, United States; 6 Mayo Clinic, Rochester, MN, United States

**Keywords:** latent dirichlet allocation, machine learning, natural language processing, recurrence, term frequency-inverse document frequency, thyroid cancer

## Abstract

**Objective:**

To evaluate the performance of machine learning (ML) models tasked with predicting thyroid cancer recurrence using natural language processing (NLP) and large language model (LLM) derived features.

**Patients and methods:**

This was a retrospective study of adult thyroid cancer patients treated at an academic medical center between January 1, 2010 through December 31, 2022. Patients were classified as having cancer recurrence or no recurrence. Novel NLP features were extracted from cytopathology and surgical pathology reports using Term Frequency–Inverse Document Frequency (TF-IDF), Latent Dirichlet Allocation (LDA), and a zero-shot large language model (LLM) classification. Five learning algorithms (Decision Tree, Gradient Boosting, Logistic Regression, Random Forest, and XGBoost) were trained to predict cancer recurrence utilizing a combination of NLP-derived features and structured electronic medical record (EMR) variables. All models were evaluated using stratified 5-fold cross-validation. Model performance was assessed using F1 score and area under the receiver operating characteristic curve (ROC-AUC). The top performing model was hyperparameter-tuned, and feature importance was evaluated using SHapley Additive exPlanations (SHAP) values and gain-based importance.

**Results:**

310 patients with differentiated thyroid cancer diagnosed on final surgical pathology were included in this study. The optimized XGBoost model incorporating EMR variables and LDA-derived features achieved the best performance, with a cross-validated F1 score of 0.647 on the training set and a held-out test F1-score of 0.631 (95% CI 0.509–0.758) and ROC-AUC of 0.741 (95% CI 0.572-0.892). SHAP analysis and gain-based feature importance identified surgical pathology topic—cervical and lateral neck lymph node dissection—as the dominant predictor of cancer recurrence.

**Conclusion:**

Various classifiers including machine learning models utilizing novel NLP features derived from unstructured pathology reports may enable improved prediction of recurrence in patients with differentiated thyroid cancer.

## Introduction

Thyroid cancer is the most common endocrine malignancy and has experienced one of the most rapid increases in incidence of any cancer in the United States, with rates rising from 4.8 to 14.5 per 100,000 between 1975 and 2016 (1). Importantly, it is the most frequently diagnosed malignancy among adolescents and young adults (AYAs) aged 15-29, contributing to a 3-4% rise in all AYA cancers diagnoses between 2007-2016 (2–4). Despite high overall survival rates, locoregional or distant metastatic disease recurrence remains the primary source of cancer-related morbidity, occurring in up to one-third of all patients (5).

Accurate prediction of thyroid cancer recurrence is therefore critical for long-term survivorship and surveillance. The 2025 American Thyroid Association (ATA) guidelines stratify patients into low, low-intermediate, intermediate-high and high-risk groups based on structured clinical-pathological features and response to treatment (6). However, in the era of precision medicine, machine learning (ML) models that integrate additional patient-specific data may offer an opportunity to further enhance thyroid cancer recurrence prediction beyond current risk stratification frameworks.

ML models have previously demonstrated strong performance across different thyroid cancer prognostic tasks, outperforming the American Joint Committee on Cancer (AJCC) staging system in predicting thyroid cancer-related mortality and achieving high accuracy in prediction of lymph node metastasis (7–9). More recently, ML approaches have been applied to thyroid cancer recurrence prediction by incorporating demographic, histopathologic, and molecular features (10–13). However, limitations of such studies include the use of labor-intensive manual extraction of clinical parameters and the exclusion of readily available unstructured clinical data, such as surgical and cytopathology reports, that may contain diagnostically relevant information not captured in structured fields.

Natural language processing (NLP) techniques have been increasingly applied to automate and scale the extraction of clinically relevant information from high-volume unstructured data (14). More recently, large language models (LLMs) have been explored for a range of clinical NLP applications, including the identification of Advance Care Planning (ACP) domains for cancer patients, generation of patient education and management advice for melanoma patients and automated calculation of clinical assessment scores such as the Glasgow Coma Scale (15–17). NLP has also been applied specifically to cancer recurrence prediction; for example, a breast cancer recurrence prediction model used rule-based NLP to extract predefined features from clinical text (18). Despite these advances, the potential of other NLP approaches to derive predictive features from unstructured clinical text for cancer recurrence remains relative underexplored.

This study proposes a novel approach that integrates structured clinical data with NLP-derived features from pathology reports into machine learning models to predict thyroid cancer recurrence. We hypothesize that models incorporating unstructured data—including narrative clinical reports—will outperform traditional approaches based on structured clinical data alone. Despite the rich diagnostic information embedded in free-text reports, this data source remains underutilized in clinical prediction modeling. By extracting and incorporating these insights, we aim to investigate its ability to enhance both model performance and clinical-decision making.

## Methods

### Patient population and data collection

This is a retrospective cohort study approved by the Institutional Review Board of Icahn School of Medicine at Mount Sinai (22-00347-CR00). Informed consent was waived due to the nature of retrospective research derived chart review. Adult patients (>18 years old) with thyroid cancer diagnosed by ICD 9 and 10 codes were identified from The Mount Sinai Data Warehouse between 2010-2022 (N = 2397). Patients with complete individual patient demographic data, laboratory data, cytopathology and surgical pathology reports were included for analysis in the study (N = 355). Patients were excluded with final surgical pathology diagnosing benign, anaplastic, medullary thyroid cancers and non-invasive follicular thyroid neoplasm with papillary-like nuclear features (NIFTP) pathologies (N = 45). Recurrence (N = 33) was defined as patients with biopsy proven differentiated thyroid cancer identified after index surgical operation. Patients who had presented at the time of surgery with metastatic disease were not considered to have recurrence (**Figure 1**). The dataset was partitioned into a training set (N = 217, including 23 patients with recurrence) and a held-out test set (N = 93, including 10 patients with recurrence) using a stratified 70:30 random split.

**Figure 1:**
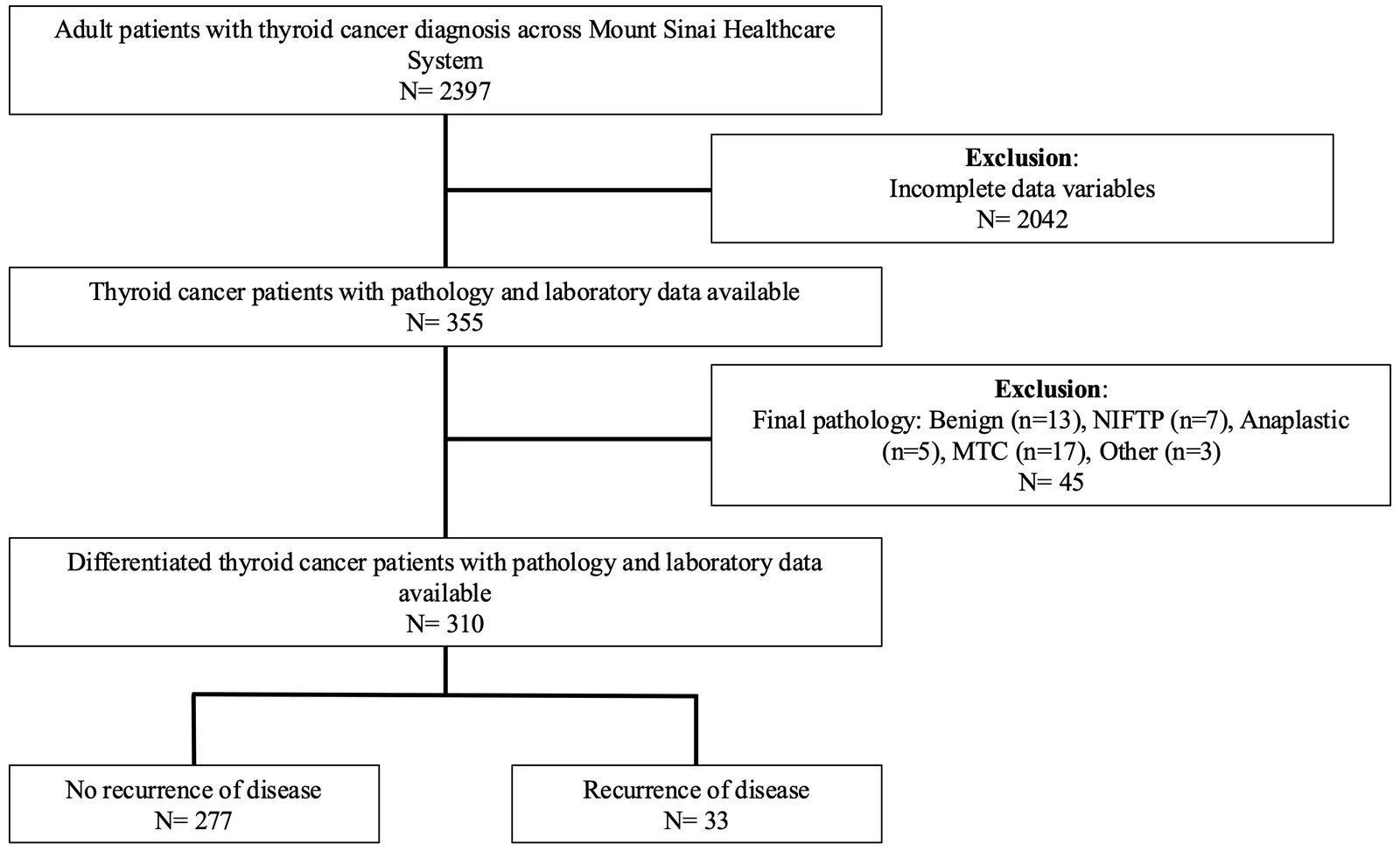
Diagram of study population inclusion and exclusion criteria. *NIFTP, Non-invasive follicular thyroid neoplasm with papillary like nuclear features *MTC, medullary thyroid cancer.

Data collection consisted of structured data (demographic features and laboratory data) and unstructured data (cytopathology and surgical pathology reports) across all known or recorded patient visits to the Mount Sinai hospital system. Data for patient race and insurance type were collapsed to form a smaller number of categories to ensure model stability during training. Patient age and BMI were defined as their age and BMI during the time of initial thyroid surgery. BMI was further collapsed by greater or equal to 25 and less than 25. For patients with multiple thyroglobulin levels, the level closest to one year after surgery was used. Cytopathology reports (from the thyroid nodule preoperative fine needle aspiration biopsy) and thyroid surgical pathology reports were collected for all patients for natural language processing and feature engineering tasks. One pathology and cytopathology report were collected for each patient’s specimen. **Figure 2** demonstrates the overall study design.

**Figure 2:**
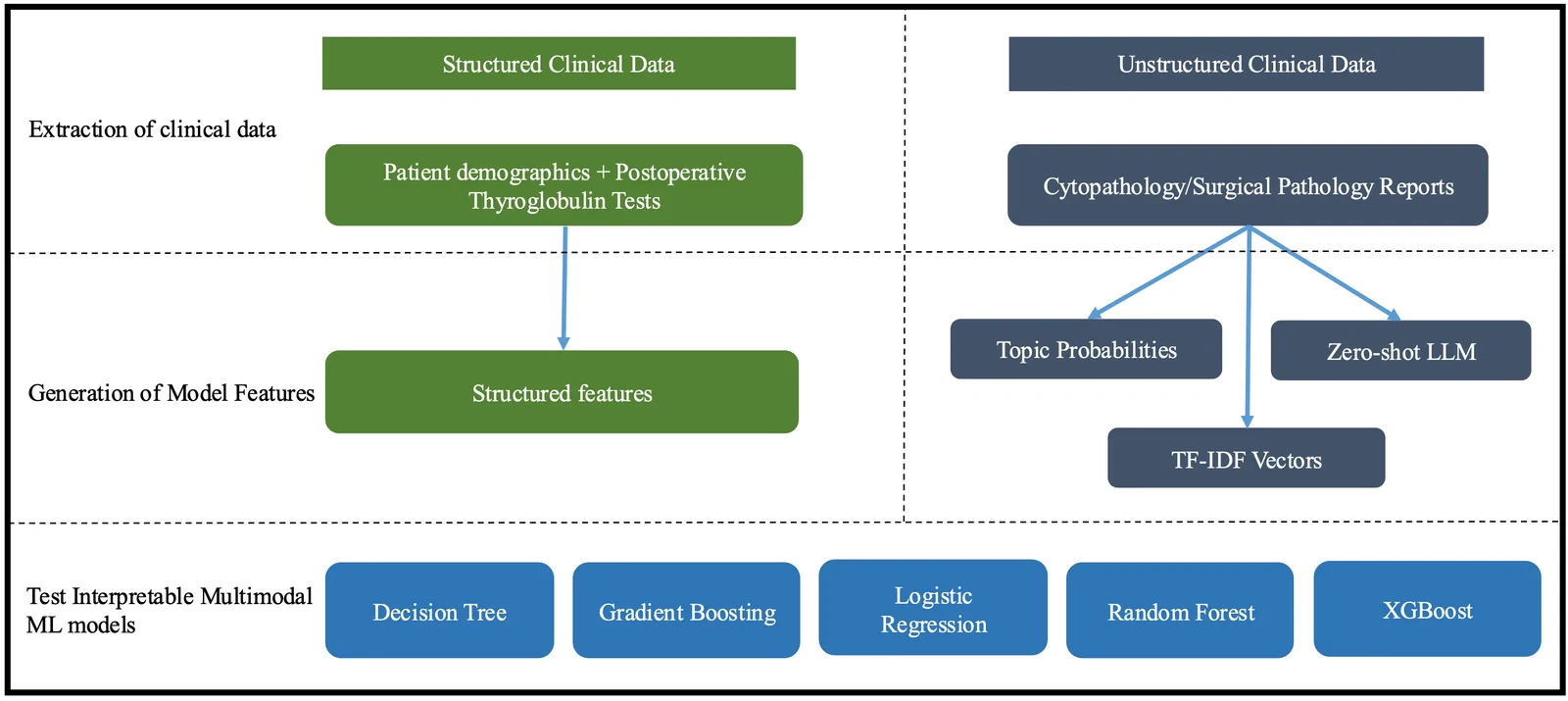
Overview of study design. Structured features include demographics and Thyroglobulin tests. Unstructured features include topic probabilities, TF-IDF, and LLM. Different combination of features were performed on all models for evaluation.

### Natural language processing

Predictive features were extracted from cytopathology and surgical pathology reports using three methods: TF-IDF, LDA and LLM. **Figure 3** illustrates the workflow for feature extraction for each NLP method.

**Figure 3:**
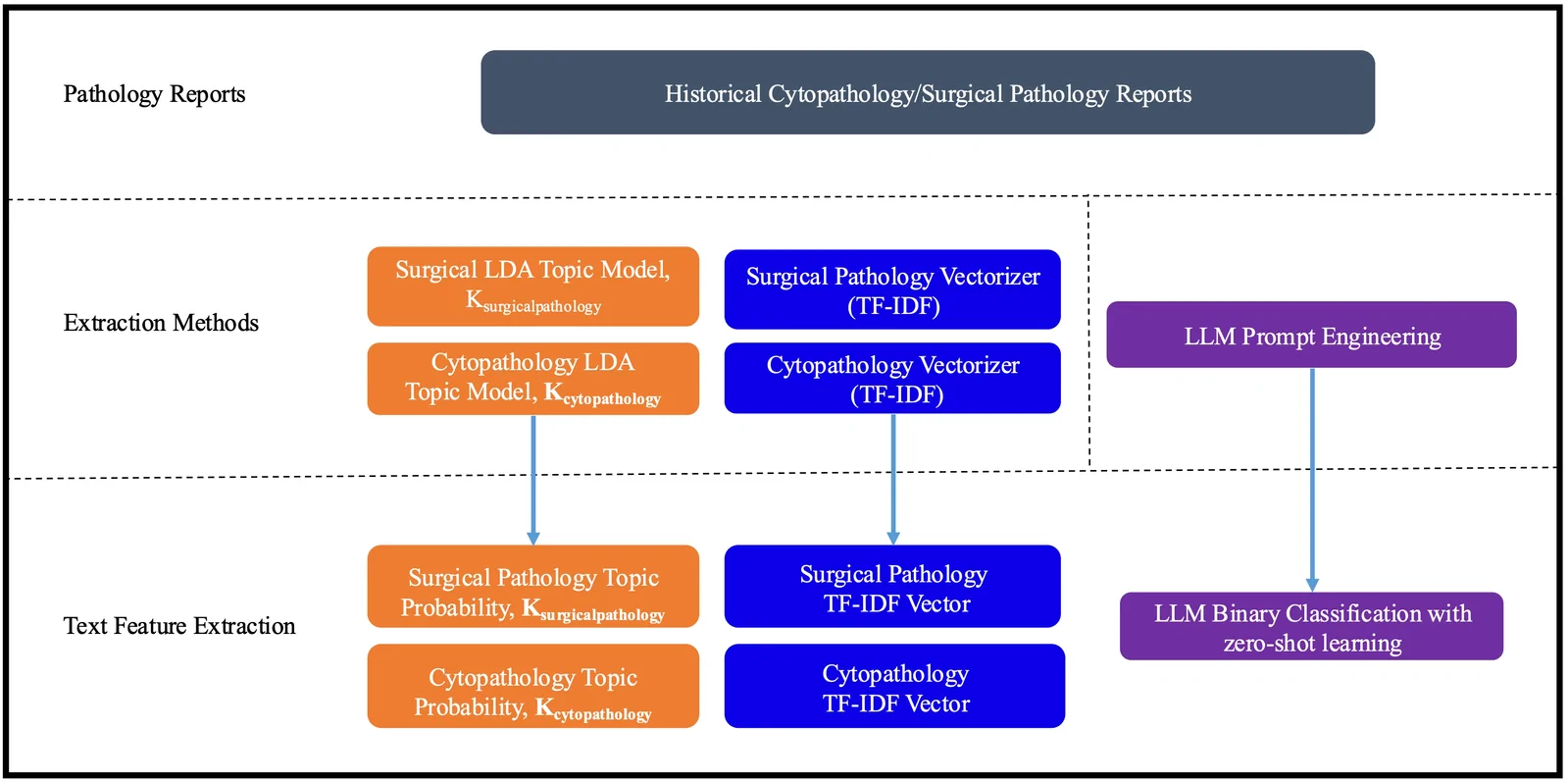
Feature extraction process for pathology reports. Coherence score optimization was performed for optimal K clusters. LDA topic probabilities and TF-IDF vectors were generated using surgical pathology and cytopathology reports. LLM classification with zero-shot learning was generated through a combination of stitched and individual reports.

#### TF-IDF features

TF-IDF is a standard statistic that indicates the importance of individual words within a body of text. It measures how often a word appears in a document while weighing for the number of documents in a collection that contains the individual words. Feature matrices were automatically computed with TfidfVectorizer from scikit-learn, with appropriate tokenizer preprocessing. Parameters for the Vectorizer can be found in **Supplementary Table 1**. The choice of top number of words to include is an adjustable hyperparameter during training; a large number results in sparse features and increases risk of overfitting due to number of features exceeding sample size (classic p >> n problem in statistics), while a conservative number results in loss of information from omitting key signal words.

#### LDA features

LDA is a type of unsupervised machine learning model that learns to identify clusters of similar words within a body of text. First proposed by Blei et al., it is a Bayesian hierarchical model that assumes each document is a finite mixture over a set of K underlying topics, and each topic is in turn distributed over clusters of individual words (19). K is a hyper-parameter that can be tuned to yield an optimal number of topics. Hence, topic probabilities are an explicit representation of themes contained in a document, and important words within each topic can be utilized to identify overall themes within a collection of documents. We utilized a novel repurposing of these topic probabilities as additional predictive features by first training a topic model over a training set of surgical pathology and cytopathology reports and identifying the optimal model with respect to hyper-parameter K. Then on the held-out test set, we applied the optimal model to extract topic probabilities for each patient’s cumulative surgical and cytopathology reports.

The standard metric for identifying the optimal K number of clusters or topics is to use coherence scores. Higher coherence scores indicate better interpretability of identified topics. Finding optimal K was done exclusively on the training set. Topics K was found to be K = 8 for surgical pathology reports and K = 5 for cytopathology reports after iterative topic inspection and removal of non-clinical noise such as administrative language and identifiers. Topic words were then evaluated by an endocrine surgeon (DTL) to generate topic themes for each K (**Tables 1**, **2**). Each topic K is a topic probability vector and was considered a feature for model development.

**Table 1:** Topic themes for cytopathology.

Topic	Theme	Top important words
Topic 1	Pathology/cytology features	cell, specimen, thyroid, smear, epithelial, nodule, interpretation, aspiration, carcinoma, bloody
Topic 2	Preparation for FNA	smear, cell, fna, specimen, nodule, needle, guide, episode, material, preliminary, aspiration, final, slide
Topic 3	Malignancy	thyroid, specimen, cell, fna, clinical, bethesda, follicular, carcinoma, category, papillary, final, malignancy
Topic 4	Malignancy	mutation, cancer, follicular, gene, cytology, neoplasm, undetermined_significance, molecular, papillary, tert
Topic 5	Preparation for FNA	needle, aspiration, thyroid, ultrasound, fine, biopsy, clinical, specimen, nodule, guidance, smear, alcohol, slide

**Table 2:** Topic themes for surgical pathology.

Topic	Theme	Top important words
Topic 1	Intraoperative biopsy	frozen, consultation, tissue, fresh, submit, parathyroid, negative, margin, positive, biopsy, mass, intraoperative
Topic 2	Cervical and lateral neck lymph node dissection	lymph, node, level, dissection, neck, tissue, carcinoma, section, metastatic, involve, tumor, papillary
Topic 3	Intraoperative biopsy	frozen, tissue, fresh, margin, proximal, consultation, fragment, cancer, evaluation, biopsy, central
Topic 4	Cervical and lateral neck lymph node dissection	lymph, node, carcinoma, level, tumor, number, dissection, extension, neck, central, metastatic
Topic 5	Cytology	cell, aspirate, needle, nuclear, cellular, aspiration, irregularity, malignant, colloid
Topic 6	Cervical and lateral neck lymph node dissection	lymph, node, dissection, number, central, extension, tumor, invasion, level, compartment, neck
Topic 7	Extent of cancer involvement	thyroid, lobe, tumor, lymph, carcinoma, invasion, node, isthmus, margin, involve, papillary
Topic 8	Extent of cancer involvement	thyroid, lobe, tumor, carcinoma, invasion, lymph, nodule, ink, node, isthmus, surface, extension, margin, papillary

#### LLM with zero-shot learning

A zero-shot learning LLM refers to a model that is capable of completing a task without prior examples or pre-training. Using Llama3.1 8B, a recurrence classification was generated as a feature (20). Stitched surgical pathology/cytopathology reports were used as inputs within the prompt. OpenAI’s best practices for prompt engineering were followed to ensure effective outcomes (21). **Supplementary Tables 2** and **3** describe the model used and an example prompt that was used to return a binary classification. The prompt was designed with three aims: adoption of an expert persona, defining the actual classification task, and minimizing hallucination by giving bounds on acceptable responses. Temperature was set to 0 to ensure consistency (22). The final LLM output is a binary recurrence label for each patient using their clinical reports as the input. The results were then evaluated against the annotated ground-truth labels.

### Model development

To evaluate the value added by text features (TF-IDF, topic modeling via LDA, LLM) to ML model prediction performance, we first trained baseline models using only EMR features. These EMR features included age at surgery, sex, race, insurance type, pre-operative BMI, family history, and postoperative thyroglobulin level. Five learning algorithms (Decision Tree, Gradient Boosting, Logistic Regression, Random Forest, and XGBoost) were evaluated with six feature configurations, totaling 30 model evaluations. All evaluations were based on the outcome of binary classification of differentiated thyroid cancer recurrence following index surgery operation. Further description of model functions used can be found in **Supplementary Table 4**. All 30 models were evaluated using a stratified 5-fold cross-validation method to preserve cohort imbalance of recurrence patients (10.6% recurrence rate). Default hyperparameters were used for all initial training. Macro F1-score was used as the primary performance metric to select the best performing model, which was subsequently hyperparameter-tuned using GridSearchCV (scikit-learn) with stratified 5-fold cross-validation strategy (23). The hyperparameter-tuned model was then evaluated on the held-out test set using a 1,000-iteration stratified bootstrap resampling for 95% confidence intervals (95% CI). Model calibration was assessed on the hyperparameter-tuned model using a calibration curve and Brier score. Feature importance was analyzed using both SHAP values and gain-based feature importance.

### Statistical analysis

All analyses were performed in Python version 3.12. For cohort characteristics, continuous variables are summarized with median and interquartile range, while categorical variables are summarized as N and percentages. Continuous variables were compared using the non-parametric Mann-Whitney-U test. Categorical variables were compared using the chi-square test and Fisher’s exact test for binary categories when cell counts fell below 5. A P<0.05 was considered statistically significant.

## Results

### Demographic and clinical variables

310 patients with differentiated thyroid cancer diagnosed on final surgical pathology were included in this study. All patients had cytopathology and surgical pathology data available for review. Of the 310 patients, 33 (10.6%) experienced disease recurrence while, 277 patients (89.4%) had no evidence of disease recurrence. Patient demographic, pre-operative clinical variables and post-operative thyroglobulin levels are summarized in **Table 3**. Median age was 35.1 in the recurrence cohort and 42.8 in the no recurrence cohort. A total of 63.6% (21/33) and 71.1% (197/277) of the recurrence and no recurrence cohorts were female, respectively. In both cohorts, most patients were white (45.5%, 15/33 recurrence cohort and 45.8%, 127/277 no recurrence cohort) and had private insurance (54.5%, 18/33 recurrence cohort and 51.6%, 143/277 no recurrence cohort). Postoperative thyroglobulin level was significantly different between the two groups, with median level 2.30 ng/mL in the recurrence group and 0.20 ng/mL in the no recurrence group based on Mann-Whitney U-test (*P=.003*).

**Table 3:** Demographic, pre and post-operative clinical variables for patients in the recurrence and no-recurrence patient cohorts.

Clinical variables	Recurrence N= 33	No recurrence N= 277	P-value
Demographic Age (y), median (IQR) Female, n (%) Race, n (%) White Black Asian Other/Unknown	35.1 (24.6–52.0) 21 (63.6%) 15 (45.5%) 3 (9.1%) 7 (21.2%) 8 (24.2%)	42.8 (29.4–54.9) 197 (71.1%) 127 (45.8%) 15 (5.4%) 49 (17.7%) 86 (31.0%)	0.150 0.492 0.729
Insurance type, n (%) Medicare Medicaid Private Other	8 (24.2) 5 (15.2) 18 (54.5) 2 (6.1)	47 (17.0) 68 (24.5) 143 (51.6) 19 (6.9)	0.560
Pre-operative BMI (kg/m^2^), n (%) <25 ≥25 Positive family history, n (%)	11 (33.3) 22 (66.7) 3 (9.1)	84 (30.3) 193 (69.7) 21 (7.6)	0.877 0.730
Labs Tg level (ng/mL), median (IQR)	2.3 (0.2–10.5)	0.2 (0.2–1.7)	0.003

Continuous variables are shown as median (IQR). We used Mann-Whitney U-test for continuous variables and Pearson Chi-square test for categorical variables comparisons between groups.

### Themes for topic modeling features

Our LDA technique generated topic words from patient’s cytopathology and surgical pathology reports. These words were then reviewed by an endocrine surgeon (DTL) and were assigned a theme that best represent the topic, as shown in **Tables 1** and **2**. Topic themes in cytopathology reports include pathology/cytopathology features, preparation for FNA and malignancy. Topic themes in surgical pathology reports include intraoperative biopsy, cervical and lateral neck lymph node dissection, cytology, and extent of cancer involvement.

### LLM recurrence prediction using zero-shot learning

The results of the LLM classification are shown in **Supplementary Table 5**. The model had a low F1-score of 0.393 and accuracy score of 0.542. The results were then translated to a binary classification that was used as an additional feature for the machine learning models.

### Model performance

The performance metrics for all models on the training set are presented in **Table 4**. The highest performing model utilizing baseline features (EMR only) was Gradient Boosting which achieved a F1-score of 0.521 and a ROC-AUC of 0.457. The XGBoost model incorporating EMR and LDA-derived features achieved the best performance overall, with a cross-validated training F1-score of 0.591 and a ROC-AUC of 0.744 in the training set. Hyperparameter tuning was then performed on this model which improved the performance to F1-score of 0.647 and AUC of 0.753. The final model was then evaluated once on the held-out test set, achieving a F1-score of 0.631 (95% CI 0.509-0.758) and AUC of 0.741 (95% CI 0.572-0.892). Calibration was suboptimal with a Brier score of 0.141, exceeding the null Brier score of 0.096. All held-out test performance metrics can be found in **Table 5**. An ROC graph was generated for the hyperparameter-tuned model, as shown in **Figure 4A**. Calibration curve was plotted in **Figure 4B**.

**Table 4:** Model performance on the training set.

Features	Model type	Accuracy	Precision	Recall	F1-score	AUC	PR-AUC
EMR	Decision Tree	0.816	0.464	0.474	0.469	0.474	0.109
EMR	Gradient Boosting	0.880	0.533	0.527	0.521	0.457	0.207
EMR	Logistic Regression	0.640	0.515	0.542	0.474	0.491	0.226
EMR	Random Forest	0.844	0.515	0.507	0.504	0.450	0.211
EMR	XGBoost	0.852	0.519	0.512	0.508	0.465	0.180
EMR-LDA	Decision Tree	0.811	0.502	0.506	0.503	0.506	0.130
EMR-LDA	Gradient Boosting	0.862	0.534	0.534	0.532	0.681	0.278
EMR-LDA	Logistic Regression	0.751	0.541	0.582	0.539	0.670	0.306
EMR-LDA	Random Forest	0.889	0.549	0.515	0.504	0.645	0.291
**EMR-LDA**	**XGBoost**	**0.875**	**0.703**	**0.564**	**0.591**	**0.744**	**0.392**
EMR-LDA-LLM	Decision Tree	0.811	0.504	0.506	0.505	0.506	0.131
EMR-LDA-LLM	Gradient Boosting	0.866	0.551	0.537	0.539	0.692	0.273
EMR-LDA-LLM	Logistic Regression	0.747	0.562	0.619	0.557	0.659	0.269
EMR-LDA-LLM	Random Forest	0.889	0.549	0.515	0.504	0.689	0.292
EMR-LDA-LLM	XGBoost	0.862	0.603	0.556	0.568	0.723	0.328
EMR-TFIDF	Decision Tree	0.848	0.507	0.531	0.518	0.531	0.132
EMR-TFIDF	Gradient Boosting	0.871	0.533	0.522	0.519	0.564	0.192
EMR-TFIDF	Logistic Regression	0.797	0.561	0.578	0.561	0.644	0.258
EMR-TFIDF	Random Forest	0.880	0.498	0.510	0.496	0.683	0.308
EMR-TFIDF	XGBoost	0.875	0.446	0.490	0.467	0.700	0.263
EMR-LDA-TFIDF	Decision Tree	0.852	0.570	0.604	0.584	0.604	0.202
EMR-LDA-TFIDF	Gradient Boosting	0.862	0.499	0.517	0.507	0.587	0.183
EMR-LDA-TFIDF	Logistic Regression	0.797	0.542	0.555	0.543	0.648	0.306
EMR-LDA-TFIDF	Random Forest	0.899	0.551	0.537	0.530	0.706	0.336
EMR-LDA-TFIDF	XGBoost	0.871	0.520	0.522	0.513	0.673	0.276
EMR-LDA-TFIDF-LLM	Decision Tree	0.843	0.568	0.581	0.574	0.581	0.152
EMR-LDA-TFIDF-LLM	Gradient Boosting	0.862	0.499	0.517	0.507	0.585	0.173
EMR-LDA-TFIDF-LLM	Logistic Regression	0.797	0.556	0.573	0.557	0.638	0.259
EMR-LDA-TFIDF-LLM	Random Forest	0.880	0.548	0.515	0.508	0.684	0.266
EMR-LDA-TFIDF-LLM	XGBoost	0.871	0.520	0.522	0.513	0.675	0.277

Bold values: Best performing model.

**Table 5:** Held-out test set performance.

Model	Accuracy (95% CI)	F1-score (95% CI)	Precision (95% CI)	Recall (95% CI)	AUC (95% CI)	PR-AUC (95% CI)	Brier score (95% CI)
XGBoost-EMR-LDA	0.817 (0.742–0.892)	0.631 (0.509–0.758)	0.616 (0.515–0.725)	0.677 (0.522–0.840)	0.741 (0.572–0.892)	0.317 (0.153–0.582)	0.141 (0.110–0.172)

**Figure 4:**
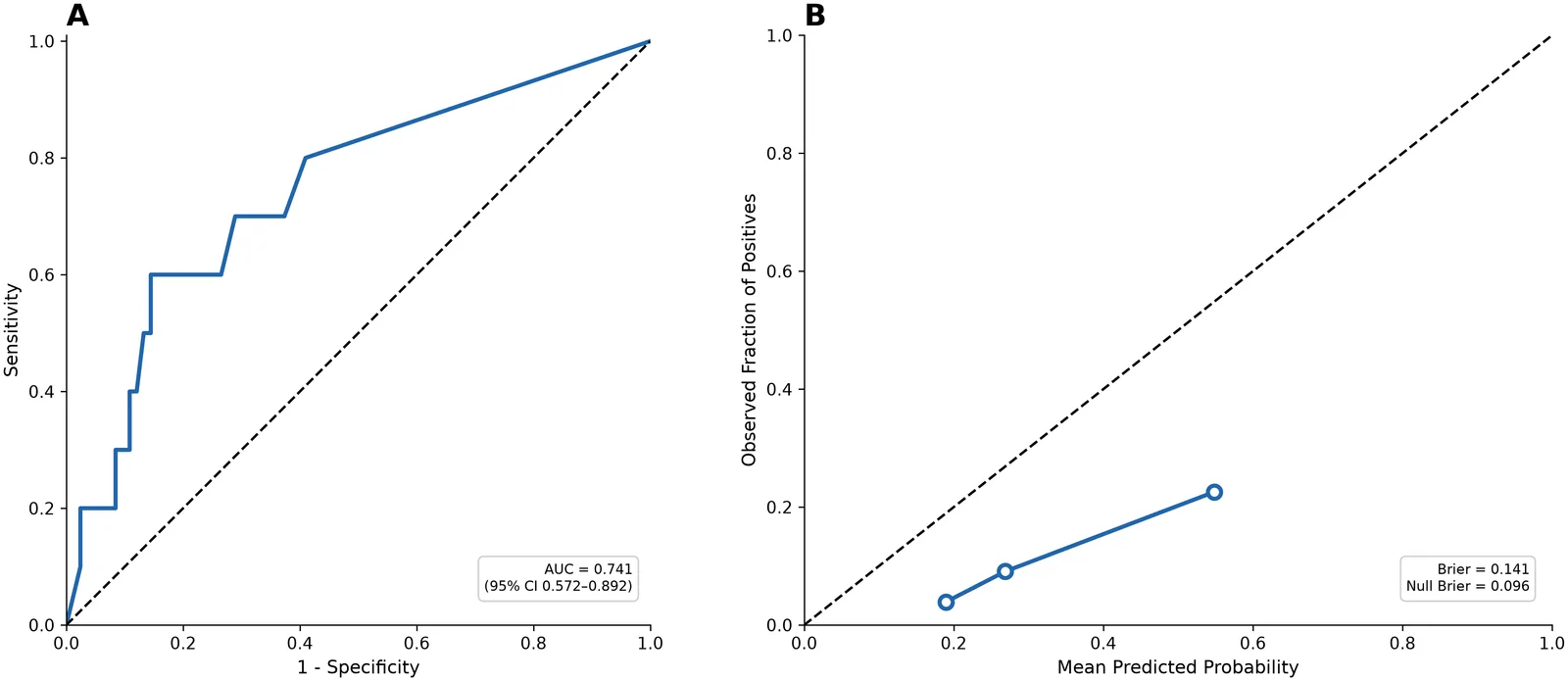
Model evaluation – test set of XGBoost model. **(A)** Receiver operating characteristic (ROC), **(B)** Calibration Curve.

### Model explainability

The gain-based feature importance graph illustrates the most important features were LDA-derived. Surgical Pathology LDA topic 2, representing the theme of cervical and lateral neck lymph node dissection, was identified as the most important feature in the final hyperparameter-tuned model. Other features were: Cytopathology LDA topic 3 (malignancy), Asian race, Surgical Pathology LDA Topic 6 (cervical and lateral neck lymph node dissection), Surgical Pathology LDA Topic 7 (extent of cancer involvement), female sex, Surgical Pathology LDA Topic 4 (cervical and lateral neck lymph node dissection), postoperative thyroglobulin level, Cytopathology LDA Topic 1 (pathology/cytopathology features), and Cytopathology LDA Topic 5 (preparation for FNA) (**Figure 5A**). The SHAP summary plot illustrates the contribution of each feature to individual model predictions (**Figure 5B**). Similar to the gain-based feature importance analysis, Surgical Pathology LDA Topic 2 (cervical and lateral neck lymph node dissection) was the most influential predictor of recurrence in the SHAP summary plot, with maximum SHAP values reaching 0.759. Positive SHAP values indicates that a feature increases the predicted probability of disease recurrence, whereas negative SHAP values indicate that a feature decreases the predicted probability of recurrence.

**Figure 5:**
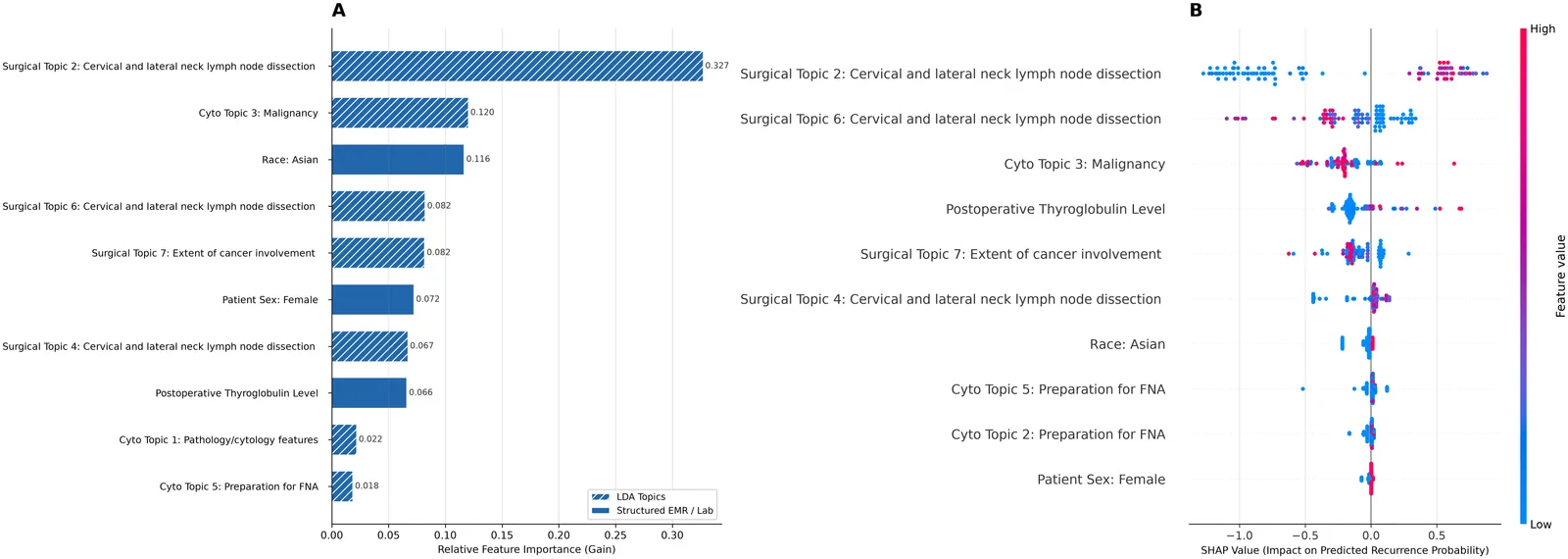
Feature importance - test set of XGBoost model. **(A)** Feature Importance (Gain) graph. **(B)** SHAP summary plot.

## Discussion

This study evaluated the performance of multi-source machine learning models for predicting thyroid cancer recurrence using surgical pathology and cytopathology pathology reports extracted from the EMR. NLP-derived features included LDA topic modeling, TF-IDF vectorization, and LLM-generated representations. The top-performing model— XGBoost classifier—integrated structured EMR data with LDA features, and was optimized through hyperparameter grid search, achieved a F1-score of 0.631 (95% CI 0.509-0.758) and AUC of 0.741 (95% CI 0.572-0.892) on the held-out test set. This performance is comparable to prior thyroid cancer recurrence models that rely solely on structured clinical variables alone, which highlights the practical advantage of NLP-derived features as a scalable alternative to labor-intensive manual data extraction (10, 24). Notably, the structured EMR-only models demonstrated limited discrimination, with AUCs ranging from 0.45-0.49, indicating the available structured EMR features did not reliably distinguish recurrence in this cohort. Given the significant difference in postoperative Tg levels between patients with and without recurrence, we additionally evaluated postoperative thyroglobulin as a clinically relevant single-variable benchmark. A thyroglobulin-only logistic regression model evaluated using the same stratified 5-fold cross validation framework achieved an AUC of 0.643, compared with an AUC of 0.744 for the combined EMR-LDA XGBoost model. The improved discrimination of the combined model relative to the thyroglobulin-only model suggests that the addition of NLP-derived feature may provide incremental predictive information beyond postoperative thyroglobulin alone. It is important to note that the optimized model demonstrated poor calibration, with a Brier score of 0.141 (null Brier score= 0.096). Although the model achieved moderate discrimination, as reflected by its F1-score and AUC, its probability estimates were not sufficiently reliable for clinical prediction of thyroid cancer. Such poor calibration indicates that the predicted probabilities may not accurately reflect patients’ absolute risk of recurrence, potentially limiting their clinical utility. Calibration may also vary across external populations with different patient characteristics and baseline recurrence rates, underscoring the need for external validation and potential recalibration before clinical implementation (25). Consequently, the current model should not be interpreted as a clinically applicable recurrence prediction tool. Rather, these findings demonstrate the feasibility and potential value of extracting informative features from free text pathology reports using NLP to enhance machine learning models.

Importantly, both the SHAP summary plot and gain-based feature importance graph demonstrated that seven of the top ten most important features were LDA-derived. The dominant topic was characterized by terms related to cervical lymph node dissection, a clinically coherent theme closely linked to nodal disease and disease extent in thyroid cancer. The emergence of this topic through an unsupervised approach demonstrates the ability of LDA to identify clinically meaningful patterns embedded within unstructured pathology narratives without requiring these features to be predefined or manually extracted. Its association with recurrence further suggests that topic modeling can capture information relevant to recurrence risk from clinical reports. However, this association may not serve as independent prognostic factor and may be a proxy for the performance of a neck dissection and the underlying clinical nodal disease and greater disease extent. Notably, postoperative thyroglobulin levels, which are biochemical markers of recurrence, emerge as top ten features for both SHAP and gain-based feature importance. This reflects the relative strength of the topic model signal compared to a single laboratory value, given the well-established role thyroglobulin as a predictor of thyroid cancer recurrence.

In contrast, both LLM-derived and TF-IDF features were excluded in the best performing model. This finding suggests that zero-shot LLM classification, without domain-specific fine tuning, may not yet capture the nuanced language patterns associated with cancer recurrence in clinical narrative text. Given the rapidly evolving capabilities of LLMs for clinical NLP tasks, this demonstrates the limitation of zero-shot performance for this specific application alone. Similarly, TF-IDF may lack the contextual understanding within clinical narrative text that topic modeling is able to achieve with topic themes.

Topic modeling is an established technique in clinical NLP, and prior studies have demonstrated the utility of topic-derived features for predictive modeling. Rumshisky et al. improved psychiatric readmission prediction by incorporating LDA features derived from discharge summaries (26) while Han et al. used LDA-derived features from clinical notes to predict complications of diabetes mellitus (27). Our study extends this body of work by applying LDA to unstructured pathology reports and incorporating the resulting topic-derived features into models of thyroid cancer recurrence. Furthermore, rather than relying on a single representation of unstructured text, we evaluated complementary NLP approaches, including LDA, TF-IDF and LLM-based classification, in combination with structured clinical data. This multimodal approach demonstrates the potential for clinically interpretable topic-derived features and other representations of pathology narratives to complement structured clinical information in cancer recurrence prediction.

Our study contained several limitations. The patient cohort was imbalanced, with recurrent cases comprising approximately 10% of the dataset. Although this incidence is consistent with the reported low recurrence rates in differentiated thyroid cancer, this imbalance may limit model generalizability and could bias the predictions. We accounted for this imbalance by relying on F1 score and ROC-AUC to identify the highest performing model, rather than using accuracy. Poor calibration score of our model may also limit generalizability. Additionally, the exclusion of many patients because of incomplete data may have introduced selection bias. This likely reflects our institution’s role as a tertiary referral center, where laboratory results and cytopathology reports frequently originate from outside facilities and are therefore not captured as discrete, extractable data within our EMR. We did not define a minimum follow-up duration for recurrence classification and data on individual follow-up were not available. Consequently, we were unable to perform time-to-event analysis or assess the potential impact of differential follow-up. Patients with shorter follow-up may have been classified as non-recurrent despite subsequently developing recurrence, potentially resulting in misclassification and underestimation of the true recurrence rate. Future studies would benefit from incorporating a time to recurrence prediction model to address this limitation. The single-institutional nature of this study and lack of external validation limit the generalizability of our findings, as differences in patient populations and practices at different institutions may affect model performance. These limitations may be addressed in future studies by aggregating multi-institutional data, which would also increase the number of patients with recurrence and enable more robust model development and evaluation. External validation in independent, multi-institutional cohorts will be necessary to assess model generalizability and reproducibility prior to evaluating its clinical utility and potential deployment. Several limitations relate to model development. Our EMR-only baseline model did not include established predictors of DTC recurrence such as AJCC/TNM stage and ATA risk category, because these variables were not readily available as structured data at our institution. The EMR-only model was intended to provide a pragmatic baseline for evaluating the incremental value of NLP-derived features. Therefore, these features may only partially capture established clinical predictors documented in unstructured text. We also did not benchmark against a model trained on manually abstracted surgical pathology data—a comparison that could better isolate the independent contribution of our approach. Additionally, only one LLM (Meta Llama3.1 8B) was evaluated, whereas testing newer or larger models may improve performance. Finally, future work should explore prompt engineering alongside supervised fine-tuning, reinforcement learning with human in the loop feedback and methods such as Low-Rank Adaptation (LORA) to further enhance LLM classification performance (28–31).

In conclusion, this study demonstrates a novel approach to incorporating NLP-derived features from unstructured narrative reports as structured inputs for machine learning cancer recurrence prediction models. We show that topic modeling can potentially extract clinically meaningful features from pathology text. This can reduce the burden of manual abstraction with large-scale clinical dataset construction. These findings support further investigation on the use of clinical narrative reports for prediction models and highlight both the current limitations of LLMs in this domain.

## Data Availability

The dataset used in this study is not publicly available due to the inclusion of protected patient health information. Requests to access the datasets should be directed to denise.lee@mountsinai.org.
