# Supplementary material for: Incorporating natural language processing-derived features from pathology reports into machine learning models to predict differentiated thyroid cancer recurrence
Source: Front Endocrinol (Lausanne). 2026 Oct 21;17:1939077. doi: 10.3389/fendo.2026.1939077 (PMC13730829; doi:10.3389/fendo.2026.1939077)
Supplement: Supplementary file 2 [file DataSheet2.docx]

**Supplementary Materials**

***Incorporating National Language Processing-Derived Features from Pathology Reports into Machine Learning Models to Predict Differentiated Thyroid Cancer Recurrence***

Contents

[Supplemental Methods	2](#_Toc234925588)

# Supplemental Methods

Supplementary Table 1. Parameters used for TF-IDF Vectorizer.

| Parameters | Value |
| --- | --- |
| min_df | 5 |
| max_df | 0.5 |
| ngram_range | (1,3) |
| max_features | 200 |
| stopwords | ENGLISH_STOP_WORDS from sklearn.feature_extraction.text + 'patient', 'history', 'report', 'noted', 'seen', 'showed', 'procedure', 'date', 'signed', 'please', 'per', 'also', 'within', 'given', 'however', 'therefore', 'would', 'could', 'should', 'may', 'might', 'shall', 'will', 'used', 'using', 'performed', 'obtained', 'identified', 'right', 'left', 'upper', 'lower', 'total', 'been', 'was', 'were', 'has', 'have', 'had', |

Supplementary Table 2. Model information. We chose an open-sourced large language model from Meta. This model is available to the general public at no cost.

| Model | Company | Parameters | Context Window |
| --- | --- | --- | --- |
| Llama3.1 | Meta | 8B | 128K |

Supplementary Table 3. Description of Zero-shot LLM example prompt. This prompt is tasking the LLM to classify cancer recurrence from the provided surgical pathology and cytopathology reports.

| **Model** | **Prompt** | **Purpose of Prompt** | **Summary** |
| --- | --- | --- | --- |
| Llama 3.1 8B | You are a doctor specializing in thyroid cancer and recurrence. Recurrence cancer refers to cancer that returns after treatment. | Expert persona | This prompt is tasking the LLM to classify cancer recurrence from the provided surgical pathology and cytopathology reports. |
|  | You will determine if there is evidence of cancer recurrence based off surgical pathology and/or cytopathology reports below. Return recurrence if there is evidence of cancer recurrence. Return no recurrence if there is no evidence of cancer recurrence. | Task |  |
|  | Do not return additional text or summary or key points or key findings | Reduce hallucination |  |

Supplementary Table 4. Model Implementation Functions

| Model Type | Library | Function |
| --- | --- | --- |
| Logistic Regression | Sklearn.linear_model | LogisticRegression() |
| Decision Tree | Sklearn.tree | DecisionTreeClassifier() |
| Random Forest | Sklearn.ensemble | RandomForestClassifier() |
| Gradient Boosting | Sklearn.ensemble | GradientBoostingClassifier() |
| XGBoost | xgboost | XGBClassifier() |

Supplementary Table 5. LLM binary classification with zero-shot learning

|  | **Recurrence**  **N= 33** | **No Recurrence**  **N= 277** |
| --- | --- | --- |
| LLM Predicted Recurrence | 7 | 116 |
| LLM Predicted No Recurrence | 26 | 161 |
